# Prevalence of musculoskeletal disorders and physiotherapy utilization in primary care – a registry-based study in Sweden

**DOI:** 10.1186/s12875-025-03130-3

**Published:** 2025-12-15

**Authors:** Elin Östlind, Caroline Larsson, Frida Eek

**Affiliations:** 1 Department of Health Sciences, Lund University, Lund, Sweden; 2 Dalby Healthcare centre, Dalby, Sweden

**Keywords:** Primary health care, Physiotherapist, Musculoskeletal disorders, Register data, Healthcare utilization

## Abstract

**Background:**

Previous research has identified a need for relevant evidence-based clinical guidelines in primary care physiotherapy. However, an essential first step is to identify the most prevalent diagnoses among patients seeking physiotherapy. Thus, this study aims to (I) identify the most prevalent musculoskeletal diagnoses and, (II) describe physiotherapy utilization and patient characteristics among patients consulting physiotherapists in Swedish primary care.

**Methods:**

This was a register-based study including all physiotherapy visits in primary care in Region Skåne, southern Sweden during 2023. Aggregated data on patient visits registered with diagnoses according to the ICD-10 classification system, chapter VIII, *Diseases of the musculoskeletal system and connective tissue* (M-codes), were retrieved from the Skåne Healthcare register and synthesized.

**Results:**

Over the course of one year, 656,938 physiotherapy visits were registered, of which 34% (221,265) were first-time visits. Of those,176,317 (73%) had a registered M-diagnosis. Low back pain was the most common diagnosis (*n* = 13,833) followed by myalgia and cervicalgia. Spinal stenosis, fibromyalgia and osteoarthritis had the highest mean number of visits ranging from 4.2 to 4.8 visits per patient. Knee osteoarthritis had the highest estimated total number of visits (*n* = 31,390). Of the fifty most prevalent diagnoses, all but one had a higher proportion of women.

**Conclusions:**

Although low back pain was the most common diagnosis, patients with knee osteoarthritis had the highest number of visits and thus utilized the most physiotherapy resources. This study provides a comprehensive overview of the 50 most common diagnoses which may aid in determining priority areas for the development of evidence-based clinical guidelines in physiotherapy primary care settings.

**Supplementary Information:**

The online version contains supplementary material available at 10.1186/s12875-025-03130-3.

## Background

Musculoskeletal disorders (MSDs) encompass different disorders and injuries affecting bones, joints, muscles and connective tissues and are one of the leading causes of disability worldwide [1, 2]. MSDs impose a significant economic and social burden on individuals, healthcare systems and society at large and the need for rehabilitation is high [3]. As life expectancy rises, the years people live with MSD-related disability are also expected to increase [4]. Consequently, the management and rehabilitation of MSD are expected to require more healthcare resources in the future [3]. Low back pain (LBP), neck pain and osteoarthritis (OA) are some of the most occurring MSD in the population worldwide [2].

Musculoskeletal complaints are a common reason for seeking primary care [5, 6]. Examination and treatment of common MSDs is generally provided in primary care and in many countries, a general practitioner (GP) conducts the first assessment. However, it has become increasingly common that physiotherapists are the first point of contact for patients with MSDs, providing initial assessment, diagnosis, and management [7, 8]. A systematic review showed that direct access to physiotherapy is safe, cost-effective and leads to higher satisfaction among patients with MSD compared to referral from a physician [8]. Consequently, physiotherapists have an important and autonomous role in prioritizing, diagnosing and treating patients with MSDs.

Physiotherapists, as all healthcare professionals, should apply evidence-based practice to ensure that patients receive the most effective and appropriate care based on best available knowledge [9]. However, previous research has shown that many physiotherapists do not apply evidence-based practice when managing patients with MSDs [10]. Lacking time and access to scientific literature were considered important barriers to assimilate scientific evidence according to a recent Swedish study on physiotherapists’ scientific approaches [11]. Other previous studies have shown similar results regarding limited access to guidelines and also reported that relevant clinical guidelines are lacking [12, 13]. To support evidence-based practice, it is important to establish an infrastructure in the healthcare system that systematically reviews literature, develop clinical practice guidelines and adapt these guidelines for local contexts [14].

Developing guidelines is a time-consuming process, and it is important to prioritize among the numerous existing diagnoses. Commonly used prioritization criteria are the prevalence and the general burden of the diagnoses [15]. To the best of our knowledge, the most common MSDs and those carrying the highest burden in physiotherapy practice in Sweden remain unidentified. Thus, this study aims to (I) identify the most prevalent musculoskeletal diagnoses and, (II) describe physiotherapy utilization and patient characteristics among patients consulting physiotherapists in Swedish primary care.

## Methods

### Design

This is a retrospective registry-based study using data from a population-based healthcare register in southern Sweden.

### Setting

Healthcare in Sweden is predominantly tax-funded and decentralized, with governance delegated to regions and municipalities. Primary care is the first line healthcare, managed at the regional level, providing medical assessment and treatment, nursing, preventive care, and rehabilitation that does not require specialized medical or technical resources or other specific expertise. It is delivered to healthcare centres by a range of professionals, including general practitioners, nurses, physiotherapists, and others. Skåne, a region in southern Sweden is one of Sweden’s 21 regions. It is the third most populous, with approximately 1.4 million inhabitants (as of December 2023), but only the tenth largest by land area, characterized by a polycentric structure with one metropolitan area, several towns, and many smaller communities. In 2024, the number of full-time physicians per 1,000 inhabitants was 3.4 in Skåne, compared with 3.2 in Sweden overall [16] and, according to data from the Swedish Social Insurance Agency, the number of sickness benefit days per insured person was 11.5 (women) and 6.6 (men) in Skåne compared to 13.4 and 7.3 days in Sweden overall [17]. In 2023, Region Skåne had 175 primary care centres in operation.

Most physiotherapists in Skåne are employed at publicly or privately driven healthcare centres, while approximately one third operate their clinics in agreement with Region Skåne. Physiotherapists in Sweden are often the first point of contact for patients seeking care for MSDs, and no referral from a GP is required.

**Fig. 1:**
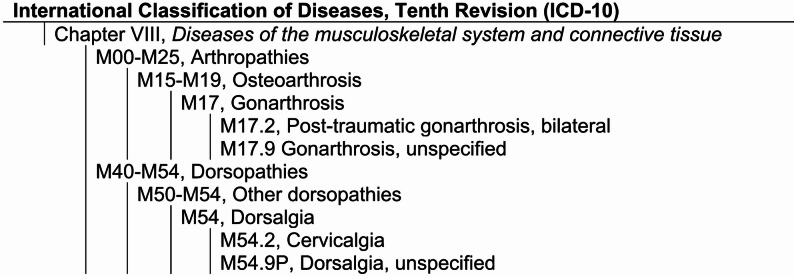
Example of hierarchical organization of the ICD-10

Diagnoses are registered for each patient encounter using the Swedish version of the International Classification of Diseases, Tenth Revision (ICD-10) [18]. ICD-10 is divided into chapters, each covering blocks with categories/sub-categories and codes representing diseases, conditions, abnormal findings or symptoms. According to the Swedish National Board of health and welfare, the primary diagnosis should be made based on “*the condition that is the primary reason for a healthcare contact*,* determined at the end of the healthcare encounter”* [19]. The primary diagnosis includes diseases, symptoms, exclusion of suspected conditions, and certain treatments when they are the reason for healthcare contact. Figure 1 shows an example of the ICD-10 structure in chapter VIII, *Diseases of the musculoskeletal system and connective tissue.*

### Data collection and management

We used the Skåne Healthcare Register (SHR) in southern Sweden to retrieve data. The SHR is an administrative healthcare register, and it holds information transferred from both the Swedish population register, computerized medical records and administrative application sources on all healthcare consultations in the Skåne region. Aggregated information on all physiotherapy in-person visits in primary care in 2023 was requested with aid from Clinical Studies Sweden, Forum South, who handled the data and provided us with descriptive, aggregated data.

The information included the total number of visits, first-time visits and proportion of the first-time visits that had a registered diagnosis (ICD-10 code). Furthermore, for *each diagnosis* with more than 10 first-time visits in 2023, we received data on the following: number of unique patients, number of first-time visits, mean age (SD), mean number of visits per patient, median number of visits per patient (with interquartile range and range, i.e., min–max visits), sex distribution, and mean age for men and women, respectively.

First-time visits and recurring visits were coded in the same manner in the register and were therefore indistinguishable. To separate these two, we defined First-time visits as: *a patient’s first consultation with a physiotherapist for a particular condition in 2023*. If the same patient received a *new diagnosis* at another visit, that visit was defined as a new visit. If the patients re-visit was registered with the same diagnosis, there had to be at least a two-month interval between to be counted as a first-time visit. The total number of visits in a cohesive treatment period for a patient is defined as: *recurring visits.* Recurring visits were classified according to the diagnosis recorded at the first-time visit with the physiotherapist. We requested aggregated data on visits with a registered diagnosis within chapter XIII, *Diseases of the musculoskeletal system and connective tissue.*

### Data synthesis

The data included information on the number of visits (recurring visits) for each three- or four-digit ICD-10 code. Aggregated patient characteristics (mean age and proportion female/male sex) were obtained for all diagnoses with at least 10 visits in 2023. We received data in Microsoft Excel on the number of *first-time visits*, sex distribution and mean age (SD) per diagnosis. Treatment series that commenced in 2022 was excluded. We also requested information on the number of patients per diagnosis and the average number of visits (recurring visits) per patient and diagnosis. Data included information on mean and median (Q1-Q2), and min-max number of visits per diagnosis. All data was merged into one document and the diagnoses were organized in descending order based on the number of first-time visits per diagnosis. A new variable (Estimated total number of visits) was created by multiplying the number of patients with the average number of visits per M-code. The fifty most prevalent M-codes were presented.

## Results

In 2023, a total of 656,938 visits with a physiotherapist were registered in primary care within Region Skåne, of which 34% (221,265) were *first-time visits.* Of the first-time visits, 90% (*n* = 199,275) were assigned an ICD-10 diagnostic code. Patient visits with an M-code accounted for 72.9% (*n* = 176,317) of all diagnosed first-time visits. In total, 588 different M-codes were used, whereof only 155 (26%) were registered on more than 100 first-time visits in 2023. Some of these diagnoses were related to symptoms such as *myalgia* or *pain in joint* and others corresponded to a specific condition such as *gonarthrosis*. 16% (*n* = 28,211) had two or more diagnoses registered at their first-time visit.

### First-time visits

The most prevalent diagnoses were LBP with 13,833 first time visits, followed by *myalgia* (9,536 visits) and c*ervicalgia* (8,433 visits). Among the ten most prevalent diagnoses, four were related to *LB*P or *neck pain*. *Spinal stenosis* had the highest mean age with 72.5 (10.3) years while *Torticollis* had the lowest mean age with 8.4 (20.0) years. In general, women had a slightly higher mean age than men. The proportion of women were higher for 49 of the 50 most prevalent diagnoses. Only *torticollis* had a higher proportion of males, and the difference was minimal (50.6% males). Of the initial visits registered with *fibromyalgia*, almost all were women (95%). *Trochanteric tendinitis* and *hallux valgus* also had a vast majority (> 80%) with women. Aggregated patient characteristics on the 50 most prevalent diagnoses (140,912 first-time visits) are presented in Table 1.

**Table 1:** Prevalence of the top 50 musculoskeletal diagnoses registered in physiotherapy primary care over one year (2023)

									Women	Men
	First-time visits^a^	Unique patients	Age (years)	Estimated total number of visits		Median number of visits		Sex distribution (women)	Age (years)	
**ICD-10 category**	(n)	(n)	Mean (SD)	Mean visits per patient	(patients x mean visits)	Median (Q1-Q3)	Min-Max	% (n)	Mean (SD)	Mean (SD)
M54.5 Low back pain	13,833	13,392	51.4 (18.8)	2.3	30,668	1 (1-2)	1-64	59 (8129)	52.0 (18.5)	50.6 (19.2)
M79.1 Myalgia	9536	9430	50.5 (19.4)	2.5	23,104	1 (1-2)	1-57	68 (6479)	51.2 (18.9)	49.1 (20.3)
M54.2 Cervicalgia	8433	8089	53.2 (17.7)	2.6	20,870	1 (1-3)	1-56	66 (5554)	53.2 (17.3)	53.3 (18.4)
M17.9 Gonarthrosis, unspecified	8418	7828	68.6 (10.7)	4.0	31,390	2 (1-4)	1-81	65 (5503)	68.4 (10.8)	69.0 (10.5)
M25.5G Pain in joint, knee	8218	8048	51.6 (19.4)	2.1	16,901	1 (1-2)	1-65	59 (4812)	52.5 (19.2)	50.3 (19.6)
M54.4 Lumbago with sciatia	8064	7777	52.9 (16.4)	2.5	19,365	1 (1-3)	1-46	59 (4726)	53.5 (16.5)	52.0 (16.3)
M54.9 Dorsalgia, unspecified	7504	7313	52.6 (21.2)	2.3	17,039	1 (1-2)	1-61	61 (4594)	53.3 (20.9)	51.4 (21.6)
M75.4 Impingement syndrome of shoulder	5595	5413	57.4 (15.5)	2.5	13,587	1 (1-3)	1-57	57 (3166)	58.4 (14.8)	56.1 (15.7)
M79.6 H Pain, unspecified in foot	5221	5110	45.8 (22.2)	1.7	8432	1 (1-2)	1-39	61 (3210)	48.0 (21.4)	42.3 (23.0)
M16.9 Coxarthrosis, unspecified	3573	3337	69.8 (10.6)	3.8	12,514	1 (1-3)	1-86	62 (2229)	70.0 (10.5)	69.4 (10.8)
M25.5B Pain in joint, unspecified in shoulder	3499	3429	58.3 (17.6)	2.2	7578	1 (1-2)	1-60	56 (1970)	60.2 (17.3)	56.0 (17.8)
M70.6 Trochanteric bursitis/ Trochanteric tendinitis	3384	3242	64.0 (14.6)	2.5	8008	1 (1-3)	1-46	83 (2812)	63.7 (14.5)	65.4 (15.2)
M75.1 Rotator cuff syndrome	3130	3051	56.6 (16.6)	2.6	7872	1 (1-3)	1-56	53 (1649)	57.7 (15.7)	55.4 (17.5)
M77.1 Lateral epicondylitis	2663	2638	49.5 (11.3)	2.6	6727	1 (1-3)	1-28	55 (1465)	49.8 (11.0)	49.2 (11.7)
M25.5 F Pain in joint, unspecified in hip	2521	2490	60.0 (18.4)	2.4	6001	1 (1-2)	1-52	64 (1617)	60.4 (17.9)	59.1 (19.4)
M54.6 Pain in thoracic spine	2306	2267	46.8 (19.1)	2.1	4806	1 (1-2)	1-66	61 (1402)	48.5 (19.2)	44.4 (18.7)
M79.6B Pain, unspecified in arm	2048	2009	55.4 (18.1)	2.1	4259	1 (1-2)	1-50	55 (1133)	56.9 (17.5)	56.5 (18.7)
M79.6G Pain, unspecified in leg	2015	1996	48.0 (22.8)	2.4	4810	1 (1-2)	1-65	60 (1205)	49.6 (22.7)	45.7 (22.8)
M72.2 Plantar fascial fibromatosis	2008	1995	51.7 (15.3)	3.0	6005	2 (1-4)	1-45	68 (1372)	51.7 (14.7)	51.8 (16.4)
M79.1 F Myalgia hip/thigh	1958	1878	55.0 (20.4)	2.1	3925	1 (1-2)	1-36	64 (1254)	56.6 (19.5)	52.2 (21.6)
M76.6 Achilles tendinitis	1941	1895	53.7 (17.6)	2.7	5079	1 (1-3)	1-36	55 (1075)	53.7 (16.8)	53.6 (18.6)
M53.1 Cervicobrachial syndrome	1898	1871	51.4 (13.9)	2.5	4584	1 (1-3)	1-48	64 (1208)	50.9 (14.1)	52.1 (13.5)
M17- Gonarthrosis [arthrosis of the knee]	1845	1753	69.3 (10.7)	3.6	6276	1 (1-3)	1-84	67 (1245)	69.5 (10.8)	69.0 (10.6)
M25.5 Pain in joint	1808	1763	53.8 (18.3)	2.2	3931	1 (1-2)	1-48	66 (1188)	54.2 (18.2)	53.1 (18.6)
M79.1B Myalgia in shoulder/upper arm	1761	1721	53.2 (18.3)	2.1	3528	1 (1-2)	1-33	61 (1066)	54.5 (17.7)	51.4 (19.1)
M77.3 Calcaneal spur	1618	1575	52.8 (15.1)	2.9	4520	2 (1-4)	1-25	71 (1152)	52.7 (14.6)	53.1 (16.2)
M19.9 Osteoarthritis, unspecified site	1605	1538	69.2 (11.2)	3.1	4814	1 (1-3)	1-54	66 (1060)	69.4 (11.0)	68.9 (11.6)
M17.1 Primary gonarthrosis, unilateral or unspecified	1532	1461	68.3 (10.7)	4.8	6940	2 (1-5)	1-68	63 (965)	68.0 (10.9)	69.0 (10.3)
M48.0 Spinal stenosis	1515	1412	72.5 (10.3)	4.2	5860	2 (1-4)	1-67	60 (902)	73.0 (10.3)	71.9 (10.4)
M25.5 H Pain in joint, unspecified in ankle/foot	1424	1384	50.2 (20.5)	1.7	2394	1 (1-2)	1-37	60 (856)	51.8 (19.7)	47.7 (21.4)
M79.6E Pain, unspecified in pelvis	1365	1351	49.8 (21.1)	1.9	2540	1 (1-2)	1-28	76 (1042)	48.0 (20.3)	55.5 (22.7)
M75.0 Adhesive capsulitis of shoulder	1326	1257	55.9 (11.0)	2.9	3683	2 (1-4)	1-56	64 (850)	55.6 (10.6)	56.4 (11.7)
M17.0 Primary gonarthrosis, bilateral	1277	1128	69.8 (10.4)	4.6	5132	2 (1-4)	1-63	59 (847)	69.1 (10.8)	71.4 (9.7)
M79.6 F Pain, unspecified in thigh	1252	1231	55.0 (22.3)	2.0	2462	1 (1-2)	1-31	58 (725)	57.1 (21.3	52.1 (23.2)
M51.1 K Intervertebral disc disorders with radiculopathy, lumbar region (sciatica)	1234	1174	53.0 (15.3)	3.8	4508	2 (1-4)	1-71	56 (688)	54.4 (15.0)	51.3 (15.5)
M79.1E Myalgia in pelvic region	1212	1172	54.3 (19.2)	2.4	2836	1 (1-2)	1-44	77 (929)	53 (18.8)	58.3 (19.9)
M79.1G Myalgia, unspecified in knee/lower leg	1203	1175	46.5 (21.1)	1.7	2009	1 (1-2)	1-31	53 (637)	47.4 (20.5)	45.5 (21.7)
M43.6 Torticollis	1092	1019	8.4 (20.0)	2.4	2405	2 (1-3)	1-15	49 (539)	10.1 (21.0)	6.6 (18.9)
M19.0B Primary arthrosis in shoulder joint	1048	951	70.6 (11.9)	3.3	3129	2 (1-3)	1-41	59 (618)	72.0 (11.9)	68.5 (11.7)
M79.7 Fibromyalgia	1032	956	53.7 (12.1)	4.2	3996	2 (1-4)	1-60	95 (980)	53.5 (12.2)	57.0 (9.5)
M62.6 Muscle wasting and atrophy, not elsewhere classified	1009	988	45.7 (21.2)	1.8	1788	1 (1-2)	1-26	55 (550)	47.2 (21.0)	43.9 (21.3)
M54.9P Dorsalgia, unspecified	919	896	53.8 (20.1)	2.4	2115	1 (1-2)	1-28	63 (583)	53.8 (20.1)	53.9 (20.1)
M16.1 Primary coxarthrosis, unilateral or unspecified	853	809	69.2 (10.9)	4.4	3527	2 (1-4)	1-72	64 (543)	69.2 (11.0)	69.2 (10.6)
M48.0 K Spinal stenosis, lumbar region	814	744	71.4 (10.6)	4.7	3489	2 (1-4)	1-70	59 (483)	71.3 (10.6)	71.6 (10.6)
M54.3 Sciatica	810	797	59.6 (16.9)	1.9	1530	1 (1-2)	1-24	57 (462)	60.4 (16.9)	58.6 (16.7)
M79.6D Pain, unspecified in hand	770	769	46.1 (19.8)	1.4	1077	1 (1-1)	1-19	59 (454)	47.8 (19.3)	43.8 (20.4)
M20.1 Hallux valgus (acquired)	731	711	54.9 (18.0)	1.5	1095	1 (1-1)	1-34	81 (595)	55.2 (17.8)	53.8 (19.1)
M53.9P Dorsopathy, unspecified	707	678	71.2 (12.9)	3.7	2515	1 (1-3.8)	1-52	63 (446)	71.8 (12.1)	70.2 (14.1)
M76.5 Patellar tendinitis	701	690	32.5 (17.9)	1.9	1318	1 (1-2)	1-21	51 (361)	30.3 (17.9)	34.7 (17.6)
M21.4 Flat foot [pes planus] (acquired)	683	669	45.1 (24.1)	1.6	1064	1 (1-1)	1-34	60 (408)	49.1 (22.4)	39.1 (25.2)

*ICD-10* International Statistical Classification of Diseases and Related Health Problems 10th Revision, *SD* Standard deviation

^a^ incl. thoracic, thoracolumbar and lumbosacral disc disorders

### Recurring visits

Patients diagnosed with *spinal stenosis*, *fibromyalgia* and *OA* had the highest number of average physiotherapy visits with 4.2–4.8 visits per patient. The highest value regarding number of visits per patient in one year was 86 visits for hip OA and 84 visits for knee OA. *Gonarthrosis*,* unspecified* had the highest estimated total number of visits (31,390 visits) and thereby utilized most physiotherapy resources. In comparison, patients with LBP had on average 2.3 visits per patient and a total estimated visit of 30,668. Diagnoses related to *unspecific pain in foot/knee*/*hand* or disorders in the foot (*hallux valgus*,* pes planus*) had the lowest mean number of visits per patient 1.4 and1.7 visits, respectively (Table 1).

## Discussion

To our knowledge, this is the first study to comprehensively identify the most prevalent MSDs, physiotherapy utilization and patient characteristics in Swedish primary care. Our findings highlight that LBP was the most common diagnosis among initial visits, while knee OA had the highest estimated total number of visits. This dual pattern underscores the burden of both acute and persistent musculoskeletal conditions in primary care physiotherapy.

Several of the diagnoses presented in this study are similar to each other and (in some cases) used interchangeably by physiotherapists. For instance, knee OA was represented four times among the 50 most prevalent diagnoses and back pain (thoracic and lumbar) with or without sciatica occurred eight times. The reasons underlying the more frequent use of certain diagnoses are unclear and fall outside the scope of this study. Numerous diagnoses presented in this study were non-specific and symptom-based such as myalgia or joint pain. This reflects the complexity of diagnosing MSDs in primary care, especially at the first visit. The results align with a Danish study showing that many patients seeking a general practitioner in primary care did not have a specific diagnosis [20]. The most common symptoms that patients reported in the Danish study were related to the musculoskeletal system.

Among the ten most common M-codes, four were related to LBP or neck pain, a result consistent with previous research showing that these conditions are among the leading causes of disability globally and a frequent reason for seeking primary healthcare [3, 5, 21]. Patients with specific LBP-conditions such as *intervertebral disc disorders* or *spinal stenosis* had on average, more recurring visits than unspecific LBP. This may be explained by the natural course of the different conditions. While most patients with nonspecific LBP improve markedly within six weeks, patients with other spinal disorders such as spinal stenosis may require a longer period of rehabilitation [22].

Our results showed that OA diagnoses had the highest mean number of recurring visits per patient. Although the chronicity of OA may be the most important factor explaining the relatively high number of visits among these patients, another explanation could be the structured care program of hip and knee OA in Sweden [23]. The program has been used for almost two decades and is well implemented and recommended in Swedish healthcare. The program is delivered in primary care and consists of group lectures that are often followed by supervised exercise for 10–12 sessions. Consequently, these national recommendations may thus explain why patients with hip and knee OA had the most visits per patient. This type of structured physiotherapy is not as widely available for other MSDs in Sweden. OA also had the highest maximum number of visits per year, with approximately 80–90 visits per patient, which is considerable. Gaining insight into whether these values represent outliers would have been valuable, but this was not possible within the scope of this study.

Of the 50 most common diagnoses that we present in this study, all but one had a higher proportion of women. A similar pattern has been reported in multiple studies including cohorts of different ages and areas of the world [24–26]. A recently published Norwegian study reported a similar pattern although they only included OA, spine pain and fibromyalgia diagnoses [26]. Tyrdal et al. also investigated if first-time visits were made with a physiotherapist or a general GP. The study showed that a higher proportion of women compared to men consulted a physiotherapist first. In this study, we did not collect data on patient visits with a GP, but considering the results of the Norwegian study, one explanation for the higher proportion of women may be that men, to a greater extent, visit a GP instead of a physiotherapist. Another explanation of the higher proportion of women may be that women seem to seek primary health care to a greater extent than men. According to the results from a large cohort-study in the Netherlands, female sex was associated with seeking primary care for somatic symptoms [27].

### Implications

Our findings demonstrated physiotherapy utilization among patients with MSDs. Such data can provide important insights for healthcare planners and policymakers, particularly since MSDs are known to account for a substantial proportion of healthcare utilization and costs [28]. Identifying the most resource-demanding patient groups may support prioritization of areas where evidence-based knowledge support and treatment pathways are most urgently needed [29]. Moreover, these metrics can be used to evaluate the impact of initiatives such as digital self-management tools or structured exercise programs over time.

### Methodological considerations

A strength in this study is the use of a large, robust healthcare register in which all physiotherapy visits in primary care are registered. Although the register only covers one region in Sweden, there is nothing to suggest that they would differ significantly from the Swedish population at large. Using a register with the ICD-10 diagnoses minimizes the risk of selection bias and increases the generalizability of the findings since ICD-10 is a system that is used worldwide. The high proportion of visits with a registered ICD-10 diagnosis is also a strength in this study.

There are several weaknesses in this study that should be considered when interpreting the findings. The primary limitation in this study is the lack of individual data and the lack of standard deviation for mean number of visits. We only retrieved aggregated data which limits the possibility for in-depths analyses and group comparison. However, the aim of this study was to provide a comprehensive overview of the most prevalent MSDs in patients visiting physiotherapists in primary care and, for this purpose, the data was considered adequate. Future studies using individual-level data could allow for additional analyses that were beyond the scope of the present study. Furthermore, the registered diagnoses in our study are only based on first-time visits with a physiotherapist but these diagnoses might have been reconsidered and changed at a follow-up visit. Hence, the non-specified diagnoses might have been less prevalent at the second visit and onward. Furthermore, the two-month interval between visits was pragmatically chosen based on clinical experience but there are most likely patients seeking care several times a year for the same condition.

We would also like to acknowledge that, in our results, there are several diagnoses that are equivalent or very similar and may be used interchangeably by clinical physiotherapists. If we had chosen to consolidate equivalent/similar diagnoses, the results would have been different. However, although some OA-diagnosed might be straightforward to combine, that is not the case for many of the other diagnoses. Consequently, we chose to present each diagnose separately for clarity. In future research, we are planning to collect individual-level data and also information about comorbidities.

## Conclusion

This study provides an overview of the fifty most prevalent diagnoses in physiotherapy primary care in southern Sweden. A vast majority of patients that visited a physiotherapist in primary care in southern Sweden sought care for MSDs. Low back pain was the most common diagnosis among first-time visits, whereas knee OA accounted for the largest utilization of physiotherapy resources. Information of the estimated physiotherapy utilization in patients with MSDs can serve as a valuable metric for healthcare planners and policymakers. For example, such data may be used to ensure that the number of physiotherapists in primary care is aligned with population needs or to determine priority areas for the development of evidence-based guidelines, or to evaluate the impact of policy interventions.

## Supplementary Information

Supplementary Material 1.

## Data Availability

The datasets used in this study are available from the corresponding author upon reasonable request.

## Abbreviations

MSD: Musculoskeletal disorders
OA: Osteoarthritis
GP: General practitioner
ICD-10: International Classification of Diseases, Tenth Revision
LBP: Low back pain

## References

[1] LeBlanc KE, LeBlanc LL. Musculoskeletal disorders. Prim Care Clin Off Pract. 2010;37(2):389–406. 10.1016/j.pop.2010.02.006 20493342

[2] Nguyen AT, Aris IM, Snyder BD, Harris MB, Kang JD, Murray M, et al. Musculoskeletal health: an ecological study assessing disease burden and research funding. Lancet Reg Health Am. 2024;29:100661. 38225979 10.1016/j.lana.2023.100661 PMC10788788

[3] Cieza A, Causey K, Kamenov K, Hanson SW, Chatterji S, Vos T. Global estimates of the need for rehabilitation based on the global burden of disease study 2019: a systematic analysis for the global burden of disease study 2019. Lancet Lond Engl. 2021;396(10267):2006–17. 10.1016/S0140-6736(20)32340-0 PMC7811204 33275908

[4] GBD 2021 Other Musculoskeletal Disorders Collaborators. Global, regional, and National burden of other musculoskeletal disorders, 1990–2020, and projections to 2050: a systematic analysis of the global burden of disease study 2021. Lancet Rheumatol. 2023;5(11):e670–82. 37927903 10.1016/S2665-9913(23)00232-1 PMC10620749

[5] Haas R, Gorelik A, Busija L, O’Connor D, Pearce C, Mazza D, et al. Prevalence and characteristics of musculoskeletal complaints in primary care: an analysis from the population level and analysis reporting (POLAR) database. BMC Prim Care. 2023;24(1):1–10. 36739379 10.1186/s12875-023-01976-z PMC9898983

[6] Keavy R, Horton R, Al-Dadah O. The prevalence of musculoskeletal presentations in general practice: an epidemiological study. Fam Pract. 2023;40(1):68–74. 35747902 10.1093/fampra/cmac055

[7] Bornhöft L, Thorn J, Svensson M, Nordeman L, Eggertsen R, Larsson MEH. More cost-effective management of patients with musculoskeletal disorders in primary care after direct triaging to physiotherapists for initial assessment compared to initial general practitioner assessment. BMC Musculoskelet Disord. 2019;20(1):186. 31043169 10.1186/s12891-019-2553-9 PMC6495522

[8] Gallotti M, Campagnola B, Cocchieri A, Mourad F, Heick JD, Maselli F. Effectiveness and consequences of direct access in physiotherapy: A systematic review. J Clin Med. 2023;12(18):5832. 37762773 10.3390/jcm12185832 PMC10531538

[9] Sackett DL, Rosenberg WM, Gray JA, Haynes RB, Richardson WS. Evidence based medicine: what it is and what it isn’t. BMJ. 1996;312(7023):71–2. 8555924 10.1136/bmj.312.7023.71 PMC2349778

[10] Zadro J, O’Keeffe M, Maher C. Do physical therapists follow evidence-based guidelines when managing musculoskeletal conditions? Systematic review. BMJ Open. 2019;9(10):e032329. 31591090 10.1136/bmjopen-2019-032329 PMC6797428

[11] Eek F, Åsenlöf P, Stigmar K. Scientific approach and attitudes among clinically working physiotherapists in Sweden -a cross sectional survey. Arch Physiother. 2023;13(1):20. 37807048 10.1186/s40945-023-00173-6 PMC10561402

[12] Gleadhill C, Bolsewicz K, Davidson SRE, Kamper SJ, Tutty A, Robson E, et al. Physiotherapists’ opinions, barriers, and enablers to providing evidence-based care: a mixed-methods study. BMC Health Serv Res. 2022;22(1):1382. 36411428 10.1186/s12913-022-08741-5 PMC9677623

[13] Ferreira RM, Martins PN, Pimenta N, Gonçalves RS. Measuring evidence-based practice in physical therapy: a mix-methods study. PeerJ. 2022;9:e12666. 35036149 10.7717/peerj.12666 PMC8740513

[14] Moore JL, Friis S, Graham ID, Gundersen ET, Nordvik JE. Reported use of evidence in clinical practice: a survey of rehabilitation practices in Norway. BMC Health Serv Res. 2018;18(1):379. 29801505 10.1186/s12913-018-3193-8 PMC5970453

[15] El-Harakeh A, Morsi RZ, Fadlallah R, Bou-Karroum L, Lotfi T, Akl EA. Prioritization approaches in the development of health practice guidelines: a systematic review. BMC Health Serv Res. 2019;19(1):692. 31615509 10.1186/s12913-019-4567-2 PMC6792189

[16] Regionfakta. Number of physicians per 1,000 inhabitants. Cited 18 Nov 2025. Available from: http://www.regionfakta.com/vastmanlands-lan/samhallets-service/Antal-lakare-per-1000-invanare/

[17] Swedish Social Insurance Agency. Sickness absence rate in 2024. Cited 18 Nov 2025. Available from: https://www.forsakringskassan.se/statistik-och-analys/statistikdatabas#!/sjuk/ohm-sjptal

[18] World Health Organization. (‎2004)‎. ICD-10: international statistical classification of diseases and related health problems : tenth revision, 2nd ed. World Health Organization. Cited 22 Mar 2025. Available from: https://iris.who.int/handle/10665/42980 https://iris.who.int/handle/10665/42980

[19] National Board of Health and Welfare. (‎2024)‎. *Guidelines for diagnostic and procedure coding with ICD-10-SE and KVÅ*. National Board of Health and Welfare. Cited 6 Oct 2025. Available from: /2024-1-8487.pdf. https://www.socialstyrelsen.se/contentassets/92931c81082740ae8a9b66078e17135a

[20] Rosendal M, Carlsen AH, Rask MT, Moth G. Symptoms as the main problem in primary care: A cross-sectional study of frequency and characteristics. Scand J Prim Health Care. 2015;33(2):91–9. 25961812 10.3109/02813432.2015.1030166 PMC4834508

[21] GBD 2021 Neck Pain Collaborators. Global, regional, and National burden of neck pain, 1990–2020, and projections to 2050: a systematic analysis of the global burden of disease study 2021. Lancet Rheumatol. 2024;6(3):e142–55. 38383088 10.1016/S2665-9913(23)00321-1 PMC10897950

[22] Zileli M, Crostelli M, Grimaldi M, Mazza O, Anania C, Fornari M, et al. Natural course and diagnosis of lumbar spinal stenosis: WFNS spine committee recommendations. World Neurosurg X. 2020;7:100073. 32613187 10.1016/j.wnsx.2020.100073 PMC7322797

[23] Thorstensson CA, Garellick G, Rystedt H, Dahlberg LE. Better management of patients with osteoarthritis: development and nationwide implementation of an Evidence-Based supported osteoarthritis Self-Management programme. Musculoskelet Care. 2015;13(2):67–75. 10.1002/msc.1085 25345913

[24] Guan SY, Zheng JX, Sam NB, Xu S, Shuai Z, Pan F. Global burden and risk factors of musculoskeletal disorders among adolescents and young adults in 204 countries and territories, 1990–2019. Autoimmun Rev. 2023;22(8):103361. 37230312 10.1016/j.autrev.2023.103361

[25] Fejer R, Ruhe A. What is the prevalence of musculoskeletal problems in the elderly population in developed countries? A systematic critical literature review. Chiropr Man Ther. 2012;20(1):31. 10.1186/2045-709X-20-31 PMC3507809 23006836

[26] Tyrdal MK, Perrier F, Røe C, Natvig B, Wahl AK, Veierød MB, et al. Musculoskeletal disorders in norway: trends in health care utilization and patient pathways: a nationwide register study. Scand J Prim Health Care. 2024;42(4):582–92. 10.1080/02813432.2024.2368848 PMC11552292 39034654

[27] Ballering AV, Olde Hartman TC, Verheij R, Rosmalen JGM. Sex and gender differences in primary care help-seeking for common somatic symptoms: a longitudinal study. Scand J Prim Health Care. 2023;41(2):132–9. 36995265 10.1080/02813432.2023.2191653 PMC10193899

[28] Power JD, Perruccio AV, Paterson JM, Canizares M, Veillette C, Coyte PC, et al. Healthcare utilization and costs for musculoskeletal disorders in Ontario, Canada. J Rheumatol. 2022;49(7):740–7. 35365584 10.3899/jrheum.210938

[29] Lentz TA, Harman JS, Marlow NM, Beneciuk JM, Fillingim RB, George SZ. Factors associated with persistently high-cost health care utilization for musculoskeletal pain. PLoS ONE. 2019;14(11):e0225125. 31710655 10.1371/journal.pone.0225125 PMC6844454

[30] WMA - The World Medical. Association-WMA Declaration of Helsinki – Ethical Principles for Medical Research Involving Human Participants. Cited 27 Jul 2025. Available from: https://www.wma.net/policies-post/wma-declaration-of-helsinki/10.1001/jama.2024.21972 39425955

