# Supplementary material for: Prevalence of musculoskeletal disorders and physiotherapy utilization in primary care – a registry-based study in Sweden
Source: BMC Prim Care. 2025 Dec 15;27:29. doi: 10.1186/s12875-025-03130-3 (PMC12853896; doi:10.1186/s12875-025-03130-3)
Supplement: Supplementary file 1 — Supplementary Material 1. [file 12875_2025_3130_MOESM1_ESM.docx]

| **Table 1.** | | | | | | | | | | | |
| --- | --- | --- | --- | --- | --- | --- | --- | --- | --- | --- | --- |
| Prevalence of the top 50 musculoskeletal diagnoses registered in physiotherapy primary care over one year (2023). | | | | | | | | | | | |
|  | | | | | | | | | **Women** | **Men** | |
|  | **First-time visits^a^** | **Unique patients** | **Age (years)** | **Estimated total number of visits** | | **Median number of visits** | | **Sex distribution (women)** | **Age (years)** | | |
| **ICD-10 category** | (n) | (n) | Mean (SD) | Mean visits per patient | (patients x mean visits) | Median (Q1-Q3) | Min-Max | % (n) | Mean (SD) | Mean (SD) | |
| M54.5  Low back pain | 13833 | 13392 | 51.4 (18.8) | 2.3 | 30668 | 1 (1-2) | 1-64 | 59 (8129) | 52.0 (18.5) | 50.6 (19.2) | |
| M79.1  Myalgia | 9536 | 9430 | 50.5 (19.4) | 2.5 | 23104 | 1 (1-2) | 1-57 | 68 (6479) | 51.2 (18.9) | 49.1 (20.3) | |
| M54.2  Cervicalgia | 8433 | 8089 | 53.2 (17.7) | 2.6 | 20870 | 1 (1-3) | 1-56 | 66 (5554) | 53.2 (17.3) | 53.3 (18.4) | |
| M17.9  Gonarthrosis, unspecified | 8418 | 7828 | 68.6 (10.7) | 4.0 | 31390 | 2 (1-4) | 1-81 | 65 (5503) | 68.4 (10.8) | 69.0 (10.5) | |
| M25.5G  Pain in joint, knee | 8218 | 8048 | 51.6 (19.4) | 2.1 | 16901 | 1 (1-2) | 1-65 | 59 (4812) | 52.5 (19.2) | 50.3 (19.6) | |
| M54.4  Lumbago with sciatia | 8064 | 7777 | 52.9 (16.4) | 2.5 | 19365 | 1 (1-3) | 1-46 | 59 (4726) | 53.5 (16.5) | 52.0 (16.3) | |
| M54.9  Dorsalgia, unspecified | 7504 | 7313 | 52.6 (21.2) | 2.3 | 17039 | 1 (1-2) | 1-61 | 61 (4594) | 53.3 (20.9) | 51.4 (21.6) | |
| M75.4  Impingement syndrome of shoulder | 5595 | 5413 | 57.4 (15.5) | 2.5 | 13587 | 1 (1-3) | 1-57 | 57 (3166) | 58.4 (14.8) | 56.1 (15.7) | |
| M79.6H  Pain, unspecified in foot | 5221 | 5110 | 45.8 (22.2) | 1.7 | 8432 | 1 (1-2) | 1-39 | 61 (3210) | 48.0 (21.4) | 42.3 (23.0) | |
| M16.9  Coxarthrosis, unspecified | 3573 | 3337 | 69.8 (10.6) | 3.8 | 12514 | 1 (1-3) | 1-86 | 62 (2229) | 70.0 (10.5) | 69.4 (10.8) | |
| M25.5B  Pain in joint, unspecified in shoulder | 3499 | 3429 | 58.3 (17.6) | 2.2 | 7578 | 1 (1-2) | 1-60 | 56 (1970) | 60.2 (17.3) | 56.0 (17.8) | |
| M70.6  Trochanteric bursitis/ Trochanteric tendinitis | 3384 | 3242 | 64.0 (14.6) | 2.5 | 8008 | 1 (1-3) | 1-46 | 83 (2812) | 63.7 (14.5) | 65.4 (15.2) | |
| M75.1  Rotator cuff syndrome | 3130 | 3051 | 56.6 (16.6) | 2.6 | 7872 | 1 (1-3) | 1-56 | 53 (1649) | 57.7 (15.7) | 55.4 (17.5) | |
| M77.1  Lateral epicondylitis | 2663 | 2638 | 49.5 (11.3) | 2.6 | 6727 | 1 (1-3) | 1-28 | 55 (1465) | 49.8 (11.0) | 49.2 (11.7) | |
| M25.5F  Pain in joint, unspecified in hip | 2521 | 2490 | 60.0 (18.4) | 2.4 | 6001 | 1 (1-2) | 1-52 | 64 (1617) | 60.4 (17.9) | 59.1 (19.4) | |
| M54.6  Pain in thoracic spine | 2306 | 2267 | 46.8 (19.1) | 2.1 | 4806 | 1 (1-2) | 1-66 | 61 (1402) | 48.5 (19.2) | 44.4 (18.7) | |
| M79.6B  Pain, unspecified in arm | 2048 | 2009 | 55.4 (18.1) | 2.1 | 4259 | 1 (1-2) | 1-50 | 55 (1133) | 56.9 (17.5) | 56.5 (18.7) | |
| M79.6G  Pain, unspecified in leg | 2015 | 1996 | 48.0 (22.8) | 2.4 | 4810 | 1 (1-2) | 1-65 | 60 (1205) | 49.6 (22.7) | 45.7 (22.8) | |
| M72.2  Plantar fascial fibromatosis | 2008 | 1995 | 51.7 (15.3) | 3.0 | 6005 | 2 (1-4) | 1-45 | 68 (1372) | 51.7 (14.7) | 51.8 (16.4) | |
| M79.1F  Myalgia hip/thigh | 1958 | 1878 | 55.0 (20.4) | 2.1 | 3925 | 1 (1-2) | 1-36 | 64 (1254) | 56.6 (19.5) | 52.2 (21.6) | |
| M76.6  Achilles tendinitis | 1941 | 1895 | 53.7 (17.6) | 2.7 | 5079 | 1 (1-3) | 1-36 | 55 (1075) | 53.7 (16.8) | 53.6 (18.6) | |
| M53.1  Cervicobrachial syndrome | 1898 | 1871 | 51.4 (13.9) | 2.5 | 4584 | 1 (1-3) | 1-48 | 64 (1208) | 50.9 (14.1) | 52.1 (13.5) | |
| M17-  Gonarthrosis [arthrosis of the knee] | 1845 | 1753 | 69.3 (10.7) | 3.6 | 6276 | 1 (1-3) | 1-84 | 67 (1245) | 69.5 (10.8) | 69.0 (10.6) | |
| M25.5  Pain in joint | 1808 | 1763 | 53.8 (18.3) | 2.2 | 3931 | 1 (1-2) | 1-48 | 66 (1188) | 54.2 (18.2) | 53.1 (18.6) | |
| M79.1B  Myalgia in shoulder/upper arm | 1761 | 1721 | 53.2 (18.3) | 2.1 | 3528 | 1 (1-2) | 1-33 | 61 (1066) | 54.5 (17.7) | 51.4 (19.1) | |
| M77.3  Calcaneal spur | 1618 | 1575 | 52.8 (15.1) | 2.9 | 4520 | 2 (1-4) | 1-25 | 71 (1152) | 52.7 (14.6) | 53.1 (16.2) | |
| M19.9  Osteoarthritis, unspecified site | 1605 | 1538 | 69.2 (11.2) | 3.1 | 4814 | 1 (1-3) | 1-54 | 66 (1060) | 69.4 (11.0) | 68.9 (11.6) | |
| M17.1  Primary gonarthrosis, unilateral or unspecified | 1532 | 1461 | 68.3 (10.7) | 4.8 | 6940 | 2 (1-5) | 1-68 | 63 (965) | 68.0 (10.9) | 69.0 (10.3) | |
| M48.0  Spinal stenosis | 1515 | 1412 | 72.5 (10.3) | 4.2 | 5860 | 2 (1-4) | 1-67 | 60 (902) | 73.0 (10.3) | 71.9 (10.4) | |
| M25.5H  Pain in joint, unspecified in ankle/foot | 1424 | 1384 | 50.2 (20.5) | 1.7 | 2394 | 1 (1-2) | 1-37 | 60 (856) | 51.8 (19.7) | 47.7 (21.4) | |
| M79.6E  Pain, unspecified in pelvis | 1365 | 1351 | 49.8 (21.1) | 1.9 | 2540 | 1 (1-2) | 1-28 | 76 (1042) | 48.0 (20.3) | 55.5 (22.7) | |
| M75.0  Adhesive capsulitis of shoulder | 1326 | 1257 | 55.9 (11.0) | 2.9 | 3683 | 2 (1-4) | 1-56 | 64 (850) | 55.6 (10.6) | 56.4 (11.7) | |
| M17.0  Primary gonarthrosis, bilateral | 1277 | 1128 | 69.8 (10.4) | 4.6 | 5132 | 2 (1-4) | 1-63 | 59 (847) | 69.1 (10.8) | 71.4 (9.7) | |
| M79.6F  Pain, unspecified in thigh | 1252 | 1231 | 55.0 (22.3) | 2.0 | 2462 | 1 (1-2) | 1-31 | 58 (725) | 57.1 (21.3 | 52.1  (23.2) | |
| M51.1K Intervertebral disc disorders with radiculopathy, lumbar region (sciatica) | 1234 | 1174 | 53.0 (15.3) | 3.8 | 4508 | 2 (1-4) | 1-71 | 56 (688) | 54.4 (15.0) | 51.3 (15.5) | |
| M79.1E  Myalgia in pelvic region | 1212 | 1172 | 54.3 (19.2) | 2.4 | 2836 | 1 (1-2) | 1-44 | 77 (929) | 53 (18.8) | 58.3 (19.9) | |
| M79.1G  Myalgia, unspecified in knee/lower leg | 1203 | 1175 | 46.5 (21.1) | 1.7 | 2009 | 1 (1-2) | 1-31 | 53 (637) | 47.4 (20.5) | 45.5 (21.7) | |
| M43.6  Torticollis | 1092 | 1019 | 8.4 (20.0) | 2.4 | 2405 | 2 (1-3) | 1-15 | 49 (539) | 10.1 (21.0) | 6.6 (18.9) | |
| M19.0B  Primary arthrosis in shoulder joint | 1048 | 951 | 70.6 (11.9) | 3.3 | 3129 | 2 (1-3) | 1-41 | 59 (618) | 72.0 (11.9) | 68.5 (11.7) | |
| M79.7  Fibromyalgia | 1032 | 956 | 53.7 (12.1) | 4.2 | 3996 | 2 (1-4) | 1-60 | 95 (980) | 53.5 (12.2) | 57.0 (9.5) | |
| M62.6  Muscle wasting and atrophy, not elsewhere classified | 1009 | 988 | 45.7 (21.2) | 1.8 | 1788 | 1 (1-2) | 1-26 | 55 (550) | 47.2 (21.0) | 43.9 (21.3) | |
| M54.9P  Dorsalgia, unspecified | 919 | 896 | 53.8 (20.1) | 2.4 | 2115 | 1 (1-2) | 1-28 | 63 (583) | 53.8 (20.1) | 53.9 (20.1) | |
| M16.1  Primary coxarthrosis, unilateral or unspecified | 853 | 809 | 69.2 (10.9) | 4.4 | 3527 | 2 (1-4) | 1-72 | 64 (543) | 69.2 (11.0) | 69.2 (10.6) | |
| M48.0K  Spinal stenosis, lumbar region | 814 | 744 | 71.4 (10.6) | 4.7 | 3489 | 2 (1-4) | 1-70 | 59 (483) | 71.3 (10.6) | 71.6 (10.6) | |
| M54.3  Sciatica | 810 | 797 | 59.6 (16.9) | 1.9 | 1530 | 1 (1-2) | 1-24 | 57 (462) | 60.4 (16.9) | 58.6 (16.7) | |
| M79.6D  Pain, unspecified in hand | 770 | 769 | 46.1 (19.8) | 1.4 | 1077 | 1 (1-1) | 1-19 | 59 (454) | 47.8 (19.3) | 43.8 (20.4) | |
| M20.1  Hallux valgus (acquired) | 731 | 711 | 54.9 (18.0) | 1.5 | 1095 | 1 (1-1) | 1-34 | 81 (595) | 55.2 (17.8) | 53.8 (19.1) | |
| M53.9P  Dorsopathy, unspecified | 707 | 678 | 71.2 (12.9) | 3.7 | 2515 | 1 (1-3.8) | 1-52 | 63 (446) | 71.8 (12.1) | 70.2 (14.1) | |
| M76.5  Patellar tendinitis | 701 | 690 | 32.5 (17.9) | 1.9 | 1318 | 1 (1-2) | 1-21 | 51 (361) | 30.3 (17.9) | 34.7 (17.6) | |
| M21.4  Flat foot [pes planus] (acquired) | 683 | 669 | 45.1 (24.1) | 1.6 | 1064 | 1 (1-1) | 1-34 | 60 (408) | 49.1 (22.4) | 39.1 (25.2) | |
|  |  |  |  |  |  |  |  |  |  | |  |
| **a** First-time visits is defined as >2 months between visits  **ICD-10** International Statistical Classification of Diseases and Related Health Problems 10th Revision;  **SD** Standard deviation  ^a^ incl. thoracic, thoracolumbar and lumbosacral disc disorders | | | | | | | | | | | |
